# Improved survival with phosphodiesterase-5 inhibitor use in men with male-predominant cancers: real-world large database study

**DOI:** 10.3389/fonc.2025.1720304

**Published:** 2025-12-10

**Authors:** Daniel Uralov, Sarah R. Harrington, Hani Samarah, Anika S. Walia, Marina Aweeda, Manal U. Mustafa, Parvesh Kumar, Andrew Briskin, Julian Jackson, Eric V. Mastrolonardo, Adam J. Luginbuhl

**Affiliations:** 1 Department of Otolaryngology – Head and Neck Surgery, Thomas Jefferson University Hospital, Philadelphia, PA, United States; 2 Sidney Kimmel Medical College, Thomas Jefferson University, Philadelphia, PA, United States; 3 Sidney Kimmel Cancer Center, Thomas Jefferson University Hospital, Philadelphia, PA, United States

**Keywords:** phosphodiesterase-5 inhibitors, overall survival, male-predominant cancers, solid tumors, large database analysis, immuno-oncology

## Abstract

**Introduction:**

Phosphodiesterase-5 (PDE5) inhibitors, commonly prescribed for erectile dysfunction, may exhibit immunomodulatory properties by reducing myeloid-derived suppressor cell activity and enhancing T-cell responses. This study evaluated the association between PDE5 inhibitor use and overall survival (OS) in male-predominant solid organ malignancies.

**Methods:**

Male-predominant solid tumors were identified using male-to-female incidence ratio ≥3:1 in SEER database: prostate, bladder, colon, esophageal, hypopharyngeal, laryngeal, tonsillar, and testicular cancers. Using the TriNetX Research Network, we compared male patients prescribed PDE5 inhibitors within 6 months of cancer diagnosis to those without PDE5 exposure. Propensity score matching (PSM) was performed on demographic, clinical, and oncologic variables. OS was assessed in a combined pan-cancer cohort, stratified by overall stage, and subgroups of included cancers. Kaplan-Meier survival analyses were conducted for 1-, 3-, and 5-year OS from diagnosis.

**Results:**

After PSM, 108,630 patients were included in each arm of the pan-cancer analysis. PDE5 inhibitor exposure was associated with significantly improved OS at 1, 3, and 5 years compared with controls (5-year OS 93.8% vs 86.6%; HR, 0.42 [95% CI, 0.41–0.44]). Stratified analyses demonstrated significantly improved OS across all four stages, with hazard ratios ranging from 0.40 to 0.57. Subgroup analyses by tumor site likewise showed statistically significant improvement in OS for every included cancer type, with hazard ratios ranging from 0.35 to 0.70.

**Conclusions:**

PDE5 inhibitor use was consistently associated with improved OS across all male-predominant cancers, including in stage-stratified analyses. These findings support further investigation into the potential role of PDE5 inhibitors in cancer outcomes.

## Introduction

The tumor microenvironment (TME) promotes immune evasion and therapeutic resistance through the activity of myeloid-derived suppressor cells (MDSCs) and tumor-associated macrophages (TAMs), which impair cytotoxic T-cell function and establish and immunosuppressive microenvironment (1–3). These cells also contribute to metabolic dysregulation via increased polyamine production driven by arginase 1 activity (4–6). Given these mechanisms, pharmacologic strategies that disrupt MDSC and TAM function are of growing interest.

Phosphodiesterase-5 (PDE5) inhibitors, originally developed for pulmonary hypertension and more widely known for treating erectile dysfunction, have been shown to possess unexpected immunomodulatory properties (7, 8). A chance clinical observation in a patient receiving the PDE5 inhibitor tadalafil suggested a significant antimyeloma response, potentially through reduction of MDSC-mediated immunosuppression (9). Preclinical studies subsequently demonstrated that PDE5 inhibitors can downregulate arginase 1 and nitric oxide synthase-2 in tumor-associated myeloid cells, leading to reduced immune suppression and increased CD8+ T-cell infiltration into tumors (10–12).

Several retrospective studies have suggested that PDE5 inhibitor use may be associated with a lower incidence of colorectal cancer (13). Furthermore, experimental models combining PDE5 inhibitors with vaccine-based or checkpoint-based immunotherapies have reported improved antitumor responses in pancreatic ductal adenocarcinoma and hepatocellular carcinoma (14, 15). Despite this growing body of preclinical and early clinical data, large real-world analyses evaluating the impact of PDE5 inhibitors on survival are lacking. In this study, we investigated the association between PDE5 inhibitor exposure and overall survival in male-predominant solid organ malignancies, using propensity score–matched data from a large real-world global database.

## Materials and methods

This study followed the Strengthening the Reporting of Observational Studies in Epidemiology (STROBE) reporting guidelines. The institutional review board of Thomas Jefferson University exempted the study from review and waived informed consent because only deidentified records were used. Data were obtained from the TriNetX Collaborative Network (TriNetX, LLC, Cambridge, MA), a real-time multicenter research platform of health care organizations across the world. It contains deidentified electronic health record (EHR) data from more than 130 million patients across diverse demographic and socioeconomic backgrounds. From this network, we queried patients in the United States. Data from 2000 through 2025 were analyzed. Race information was obtained from institutional EHRs and categorized as Asian, American Indian or Alaska Native, Black or African American, Native Hawaiian or Other Pacific Islander, White, or unknown. Ethnicity was coded as Hispanic or Latino, not Hispanic or Latino, or unknown/not reported, based on standardized EHR data.

### Study population

This epidemiologic investigation used data from 103 healthcare organizations in the United States between 2000 and 2025. International Statistical Classification of Diseases, Tenth Revision (ICD-10) codes were used to define cancer type and patient characteristics. Male-predominant solid organ cancers were defined as malignancies with a male-to-female incidence ratio ≥3:1 in the Surveillance, Epidemiology, and End Results (SEER) database and were confirmed to show similar sex distribution patterns within the TriNetX dataset. This threshold was selected to capture cancers with clear male predominance while maintaining sufficient cohort size for a representative pan-cancer analysis. The cancer types included were prostate, bladder, colon, esophageal, hypopharyngeal, laryngeal, tonsillar, and testicular. Eligible patients were male, aged 18 years or older, with a primary diagnosis of one of the abovementioned cancers. We restricted the cohort to adult males to reduce sex- and indication-related confounding and to ensure adequate exposure prevalence, as PDE5 inhibitors are prescribed primarily to men for erectile dysfunction/benign prostatic hyperplasia. The exposure cohort included patients prescribed a PDE5 inhibitor (avanafil, sildenafil, tadalafil, or vardenafil) within 6 months before or after initial cancer diagnosis. The control cohort comprised patients with no record of PDE5 inhibitor prescription. Patients without documented survival follow-up or complete oncologic staging information were excluded.

### Outcome variables

The primary outcome was overall survival (OS), defined from the date of first cancer diagnosis and evaluated at 1, 3, and 5 years. To account for potential confounders, OS was analyzed in three ways: (1) in a combined pan-cancer cohort, (2) stratified by overall stage, and (3) subgroups of individual cancer types. Propensity score matching (PSM) was performed to further balance key demographic, clinical, and oncologic variables that could influence survival for all three analyses. Propensity score matching was repeated independently for each stage-stratified and cancer-specific analysis to ensure balanced covariate distributions within these subsets. Finally, to contextualize survival outcomes, the 5-year OS for each cancer type was evaluated in all TriNetX patients.

### Statistical analysis

Descriptive statistics were used to summarize baseline demographic and clinical characteristics. Continuous variables were reported as mean (range), and categorical variables as frequency (percentage). PSM was conducted in a 1:1 ratio using nearest-neighbor matching without replacement and a caliper size of 0.1 pooled standard deviations. Patients were matched based on ICD‐10 codes for age at diagnosis, race, ethnicity, cardiovascular, circulatory and respiratory diseases, type 2 diabetes, tobacco use, alcohol use disorders, and included cancers (**Tables 1**, **2**). Covariate balance after matching was assessed using standardized differences. Kaplan–Meier survival analyses were performed to estimate OS, and survival curves were compared using the log-rank test. Hazard ratios (HRs) and 95% confidence intervals (CIs) were calculated using Cox proportional hazards regression models. All analyses were performed using the TriNetX Analytics platform.

**Table 1:** Phosphodiesterase-5 inhibitors exposed cohort baseline characteristics for all cancers combined and stratified by overall stage, after propensity score matching (PSM).

Description	PDE5-i all cancers N = 108,630	PDE5-i stage I N = 425	PDE5-i stage II N = 794	PDE5-i stage III N = 326	PDE5-i stage IV N = 189
Age at index (mean ± SD)	64.1 ± 8.4	62.1 ± 8.1	64.0 ± 7.6	62.9 ± 8.1	63.2 ± 9.1
White	77,287 (71.1%)	296 (69.6%)	540 (68.0%)	216 (66.3%)	121 (64.0%)
Black or African American	17,781 (16.4%)	85 (20.0%)	164 (20.7%)	66 (20.2%)	48 (25.4%)
Asian	2,234 (2.1%)	10 (2.4%)	10 (1.3%)	10 (3.1%)	10 (5.3%)
Unknown Race	8,037 (7.4%)	33 (7.8%)	65 (8.2%)	32 (9.8%)	13 (6.9%)
Not Hispanic or Latino	85,219 (78.4%)	347 (81.6%)	633 (79.7%)	249 (76.4%)	149 (78%)
Hispanic or Latino	4,100 (3.8%)	16 (3.8%)	35 (4.4%)	18 (5.5%)	10 (5.3%)
Unknown Ethnicity	19,311 (17.8%)	62 (14.6%)	126 (15.9%)	59 (18.1%)	30 (15.9%)
Circulatory system diseases	55,338 (50.9%)	219 (51.5%)	355 (44.7%)	169 (51.8%)	107 (56.6%)
Type 2 diabetes mellitus	15,217 (14.0%)	46 (10.6%)	121 (15.2%)	65 (19.9%)	36 (19.0%)
Tobacco use	2,198 (2.0%)	11 (2.6%)	13 (1.6%)	10 (3.1%)	10 (5.3%)
Alcohol related disorders	3,707 (3.4%)	13 (3.1%)	25 (3.1%)	18 (5.5%)	10 (5.3%)
Ischemic heart diseases	13,551 (12.5%)	54 (12.7%)	104 (13.1%)	46 (14.1%)	30 (15.9%)
Respiratory system diseases	33,900 (31.2%)	139 (32.7%)	197 (24.8%)	106 (32.5%)	62 (32.8%)

All post-matching standardized mean differences were <0.1, consistent with adequate covariate balance across matched cohorts. PDE-5-i, phosphodiesterase-5 inhibitors; SD, standard deviation.

**Table 2:** Control cohort baseline characteristics for all cancers combined and stratified by overall stage, after propensity score matching (PSM).

Description	Controls all cancers N = 108,630	Controls stage I N = 425	Controls stage II N = 794	Controls stage III N = 326	Controls stage IV N = 189
Age at index (mean ± SD)	64.0 ± 8.6	64.1 ± 11.3	65.1 ± 10.8	64.6 ± 12.6	64.9 ± 10.5
White	77,484 (71.3%)	312 (73.4%)	582 (73.3%)	226 (69.3%)	128 (67.7%)
Black or African American	17,647 (16.2%)	67 (15.8%)	147 (18.5%)	55 (16.9%)	43 (22.8%)
Asian	2,300 (2.1%)	10 (2.4%)	10 (1.3%)	10 (3.1%)	10 (5.3%)
Unknown race	7,945 (7.3%)	35 (8.2%)	45 (5.7%)	35 (10.7%)	13 (6.9%)
Not Hispanic or Latino	85,386 (78.6%)	358 (84.2%)	664 (83.6%)	245 (75.2%)	158 (83.6%)
Hispanic or Latino	3,938 (3.6%)	12 (2.8%)	28 (3.5%)	17 (5.2%)	10 (5.3%)
Unknown ethnicity	19,306 (17.8%)	55 (12.9%)	102 (12.8%)	64 (19.6%)	26 (13.8%)
Circulatory system diseases	55,131 (50.8%)	232 (54.6%)	424 (53.4%)	196 (60.1%)	116 (61.4%)
Type 2 diabetes mellitus	14,907 (13.7%)	48 (11.3%)	135 (17.0%)	62 (19.0%)	41 (21.7%)
Tobacco use	1,960 (1.8%)	10 (2.4%)	14 (1.8%)	10 (3.1%)	13 (6.9%)
Alcohol related disorders	3,479 (3.2%)	10 (2.4%)	28 (3.5%)	24 (7.4%)	10 (5.3%)
Ischemic heart diseases	13,437 (12.4%)	73 (17.2%)	112 (14.1%)	56 (17.2%)	40 (21.2%)
Respiratory system diseases	33,753 (31.1%)	152 (35.8%)	244 (30.7%)	104 (31.9%)	65 (34.4%)

All post-matching standardized mean differences were <0.1, consistent with adequate covariate balance across matched cohorts. SD, standard deviation.

## Results

### All cancers

**Figure 1:**
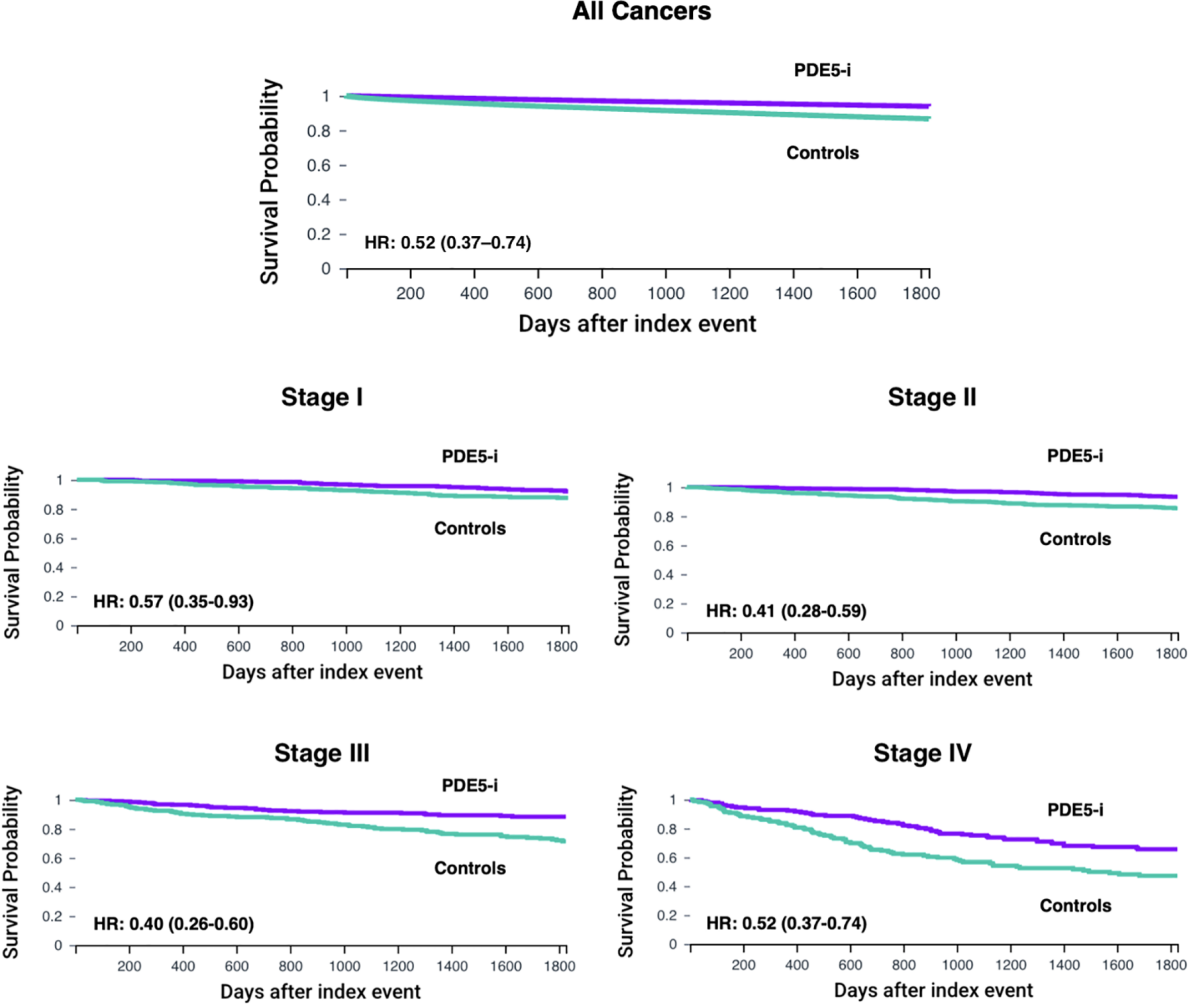
Kaplan–Meier curves for overall survival (OS) up to 5 years in male patients with male-predominant cancers, displaying all cancers combined and stratified by overall stage. OS is shown for phosphodiesterase-5 inhibitor exposed (PDE5-i) group versus controls, after propensity score matching (PSM). Hazard ratios (HR) and 95% confidence intervals (CI) are provided for each cancer type.

Before propensity score matching (PSM), the PDE5 inhibitors exposed cohort included 109,830 patients, and the control cohort included 1,393,522 patients. After PSM, 108,630 patients were included in each cohort. No significant differences in age, demographics, comorbid conditions and distribution of included cancers were observed between the two groups after matching (**Tables 1**, **2**). In the overall pan-cancer analysis of male‐predominant solid organ malignancies, PDE5 inhibitor exposure was associated with significantly improved OS at all measured timepoints compared with controls (1‐year OS 98.6% vs 95.7%, p<0.001; 3‐year OS 96.1% vs 90.7%, p<0.001; 5‐year OS 93.8% vs 86.6%, p<0.001; HR, 0.42 [95% CI, 0.41–0.44]; **Figure 1**, **Table 3**).

**Table 3:** Overall survival in patients prescribed PDE5 inhibitors compared with controls for all male-predominant cancers.

Time point	HR (95% CI)	Survival probability	Log-rank test p value
1-year	–	98.6% vs. 95.7%	<0.001
3-year	–	96.1% vs. 90.7%	<0.001
5-year	0.42 (0.41–0.44)	93.8% vs. 86.6%	<0.001

HR, hazard ratio; CI, confidence interval.

### All cancers stratified by overall stage

Staging data was available for 2,626 patients in the PDE5 inhibitor cohort and 23,983 patients in the control cohort. Post‐PSM, each cohort included 429, 794, 326 and 189 patients for Stage I, II, III and IV, respectively (**Tables 1**, **2**). For Stage I cancers, PDE5 inhibitor exposure was associated with significantly improved 3- and 5-year OS compared with controls (1-year OS not measurable; 3‐year OS 96.1% vs 92.6%, p=0.03; 5‐year OS 92.1% vs 87.6%, p=0.02; HR, 0.57 [95% CI, 0.35–0.93]; **Figure 1**). For Stage II cancers, PDE5 inhibitor exposure was also associated with significantly improved 3- and 5-year OS compared with controls (1-year OS not measurable; 3‐year OS 97.0% vs 90.5%, p<0.001; 5‐year OS 93.3% vs 85.5%, p<0.001; HR, 0.41 [95% CI, 0.28–0.59]; **Figure 1**). For Stage III cancers, PDE5 inhibitor exposure was associated with significantly improved OS at all timepoints compared with controls (1-year OS 96.4% vs 92.9%, p=0.05; 3‐year OS 91.7% vs 82.0%, p=0.001; 5‐year OS 88.3% vs 71.4%, p<0.001; HR, 0.40 [95% CI, 0.26–0.60]; **Figure 1**). For Stage IV cancers, PDE5 inhibitor exposure was also associated with significantly improved OS at all timepoints compared with controls (1-year OS 92.8% vs 82.4%, p=0.004; 3‐year OS 75.1% vs 56.5%, p<0.001; 5‐year OS 65.6% vs 47.2%, p<0.001; HR, 0.52 [95% CI, 0.37–0.74]; **Figure 1**).

### Prostate cancer

**Figure 2:**
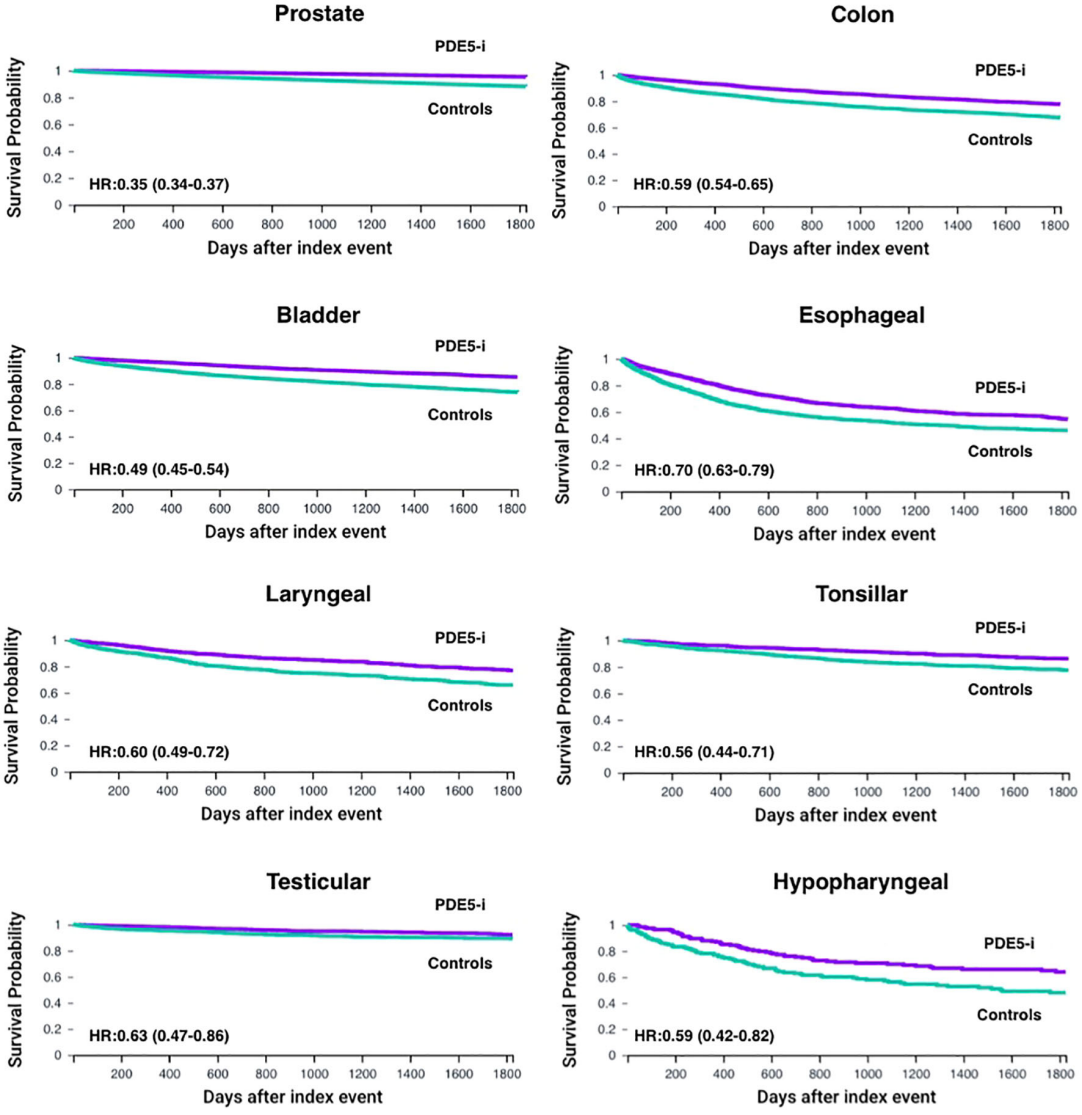
Kaplan–Meier curves for overall survival (OS) up to 5 years in male patients with male-predominant cancers, displaying individual cancer types. OS is shown for phosphodiesterase-5 inhibitor exposed (PDE5-i) group versus controls, after propensity score matching (PSM) for age, demographics and potential clinical confounders. Hazard ratios (HR) and 95% confidence intervals (CI) are provided for each cancer type.

Post‐PSM, the PDE5 inhibitor and control cohorts each included 99,010 patients with prostate cancer (**Tables 4**, **5**). PDE5 inhibitor exposure was associated with significantly improved OS at all timepoints compared with controls (1‐year OS 99.2% vs 96.7%, p<0.001; 3‐year OS 97.3% vs 92.0%, p<0.001; 5‐year OS 95.3% vs 88.1%, p<0.001; HR, 0.35 [95% CI, 0.34–0.37]; **Figure 2**). The 5-year OS of the entire TriNetX prostate cancer cohort is 85.5% (N = 1,074,022).

**Table 4:** Phosphodiesterase-5 inhibitors exposed cohort baseline characteristics for each cancer type, after propensity score matching (PSM).

Description	PDE5-i prostate N = 99,010	PDE5-i colon N = 5,463	PDE5-i bladder N = 7,852	PDE5-i esophageal N = 1,671	PDE5-i laryngeal N = 1,116	PDE5-i tonsillar N = 189	PDE5-i testicular N = 1,326	PDE5-i hypopharyngeal N = 213
Age at index (mean ± SD)	64.2 ± 7.9	63.1 ± 10.9	67.1 ± 9.6	65.6 ± 9.4	65.6 ± 8.7	62.1 ± 8.5	52.9 ± 13.3	64.6 ± 8.4
White	69,824 (70.5%)	3,844 (70.4%)	6,271 (79.9%)	1,374 (82.2%)	785 (70.3%)	956 (78.9%)	1,037 (78.2%)	152 (71.4%)
Black or African American	16,879 (17.1%)	955 (17.5%)	744 (9.45%)	140 (8.4%)	215 (19.3%)	126 (10.4%)	110 (8.3%)	43 (20.2%)
Asian	2,109 (2.1%)	119 (2.2%)	109 (1.4%)	16 (1.0%)	10 (0.9%)	10 (0.8%)	23 (1.7%)	10 (4.7%)
Unknown Race	7,225 (7.3%)	344 (6.3%)	528 (6.7%)	95 (5.7%)	73 (6.5%)	85 (7.0%)	99 (7.5%)	12 (5.6%)
Not Hispanic or Latino	78,102 (78.9%)	4,155 (76.1%)	6,162 (78.5%)	1,282 (76.7%)	862 (77.2%)	936 (77.3%)	1,013 (76.4%)	174 (81.7%)
Hispanic or Latino	3,671 (3.7%)	273 (5.0%)	238 (3.0%)	59 (3.5%)	44 (3.9%)	41 (3.4%)	72 (5.4%)	10 (4.7%)
Unknown Ethnicity	17,237 (17.4%)	1,035 (18.9%)	1,452 (18.5%)	330 (19.8%)	210 (18.8%)	234 (19.3%)	241 (18.2%)	34 (15.9%)
Circulatory system diseases	42,073 (42.5%)	3,282 (60.1%)	4,455 (56.7%)	958 (57.3%)	653 (58.5%)	622 (51.4%)	552 (41.6%)	159 (74.6%)
Type 2 diabetes mellitus	11,137 (11.2%)	1,249 (22.9%)	1,433 (18.3%)	346 (20.7%)	172 (15.4%)	167 (13.8%)	179 (13.5%)	33 (15.5%)
Tobacco use	1,624 (1.6%)	210 (3.8%)	288 (3.7%)	70 (4.2%)	95 (8.5%)	51 (4.21%)	37 (2.8%)	24 (11.7%)
Alcohol related disorders	2,875 (2.9%)	326 (6.0%)	345 (4.4%)	107 (6.4%)	121 (10.8%)	88 (7.3%)	49 (3.7%)	48 (22.5%)
Ischemic heart diseases	10,410 (10.5%)	1,006 (18.4%)	1,474 (18.8%)	314 (18.8%)	213 (19.1%)	166 (13.7%)	127 (9.6%)	43 (20.1%)
Respiratory system diseases	27,430 (27.7%)	2,192 (40.1%)	2,874 (36.6%)	703 (42.1%)	650 (58.2%)	565 (46.7%)	404 (30.5%)	155 (72.8%)

All post-matching standardized mean differences were <0.1, consistent with adequate covariate balance across matched cohorts. PDE-5-i, phosphodiesterase-5 inhibitors; SD, standard deviation.

**Table 5:** Control cohort baseline characteristics for each cancer type, after propensity score matching (PSM).

Description	Controls prostate N = 99,010	Controls colon N = 5,463	Controls bladder N = 7,852	Controls esophageal N = 1,671	Controls laryngeal N = 1,116	Controls tonsillar N = 1,211	Controls testicular N = 1,326	Controls hypopharyngeal N = 213
Age at index (mean ± SD)	64.2 ± 7.9	63.1 ± 11.1	67.1 ± 9.8	65.7 ± 9.9	65.6 ± 8.8	62.3 ± 8.6	53.1 ± 14.0	63.8 ± 9.2
White	69,981 (70.7%)	3,868 (70.8%)	6,316 (80.4%)	1,398 (83.7%)	803 (72.0%)	976 (80.6%)	1,058 (79.8%)	162 (76.1%)
Black or African American	16,827 (17.1%)	946 (17.3%)	723 (9.2%)	139 (8.3%)	211 (18.9%)	120 (9.9%)	95 (7.2%)	38 (17.8%)
Asian	2,138 (2.2%)	116 (2.1%)	99 (1.3%)	15 (0.9%)	10 (0.9%)	10 (0.8%)	26 (2.0%)	10 (4.7%)
Unknown Race	7,135 (7.2%)	342 (6.3%)	520 (6.6%)	86 (5.1%)	65 (5.8%)	77 (6.4%)	94 (7.1%)	10 (4.7%)
Not Hispanic or Latino	78,183 (79.0%)	4,169 (76.3%)	6,182 (78.7%)	1,286 (77.0%)	868 (77.8%)	935 (77.2%)	1,014 (76.5%)	178 (83.6%)
Hispanic or Latino	3,624 (3.7%)	278 (5.1%)	228 (2.9%)	48 (2.9%)	35 (3.1%)	38 (3.1%)	76 (5.7%)	10 (4.7%)
Unknown Ethnicity	17,203 (17.4%)	1,016 (18.6%)	1,442 (18.4%)	337 (20.2%)	213 (19.1%)	238 (19.7%)	236 (17.8%)	30 (14.1%)
Circulatory system diseases	42,036 (42.5%)	3,267 (59.8%)	4,444 (56.6%)	960 (57.5%)	652 (58.4%)	620 (51.2%)	558 (42.1%)	157 (73.7%)
Type 2 diabetes mellitus	11,073 (11.2%)	1,214 (22.2%)	1,410 (18.0%)	321 (19.2%)	164 (14.7%)	162 (13.4%)	161 (12.1%)	23 (10.8%)
Tobacco use	1,479 (1.5%)	192 (3.5%)	245 (3.1%)	66 (4.0%)	85 (7.6%)	48 (4.0%)	31 (2.3%)	23 (10.8%)
Alcohol related disorders	2,754 (2.8%)	299 (5.5%)	331 (4.2%)	102 (6.1%)	121 (10.8%)	73 (6.0%)	34 (2.6%)	37 (17.4%)
Ischemic heart diseases	10,393 (10.5%)	980 (17.9%)	1,458 (18.6%)	311 (18.6%)	215 (19.3%)	164 (13.5%)	123 (9.3%)	41 (19.3%)
Respiratory system diseases	27,366 (27.6%)	2,189 (40.1%)	2,859 (36.4%)	689 (41.2%)	655 (58.7%)	568 (46.9%)	399 (30.1%)	155 (72.8%)

All post-matching standardized mean differences were <0.1, consistent with adequate covariate balance across matched cohorts. SD, standard deviation.

### Colon cancer

Post‐PSM, the PDE5 inhibitor and control cohorts each included 5,463 patients with colon cancer (**Tables 4**, **5**). PDE5 inhibitor exposure was associated with improved OS at all timepoints compared with controls (1‐year OS 93.4% vs 86.9%, p<0.001; 3‐year OS 84.1% vs 75.3%, p<0.001; 5‐year OS 77.7% vs 67.4%, p<0.001; HR, 0.59 [95% CI, 0.54–0.65]; **Figure 2**). The 5-year OS of the entire TriNetX colon cancer male cohort is 68.1% (N = 229,821).

### Bladder cancer

Post‐PSM, the PDE5 inhibitor and control cohorts each included 7,852 patients with bladder cancer (**Tables 4**, **5**). PDE5 inhibitor exposure was associated with improved OS at all timepoints compared with controls (1‐year OS 96.3% vs 90.7%, p<0.001; 3‐year OS 90.2% vs 80.8%, p<0.001; 5‐year OS 85.3% vs 73.8%, p<0.001; HR, 0.49 [95% CI, 0.45–0.54]; **Figure 2**). The 5-year OS of the entire TriNetX bladder cancer male cohort is 72.7% (N = 219,836).

### Esophageal cancer

Post‐PSM, the PDE5 inhibitor and control cohorts each included 1,671 patients with esophageal cancer (**Tables 4**, **5**). PDE5 inhibitor exposure was associated with improved OS at all timepoints compared with controls (1‐year OS 81.5% vs 69.8%, p<0.001; 3‐year OS 62.6% vs 51.2%, p<0.001; 5‐year OS 54.5% vs 46.0%, p<0.001; HR, 0.70 [95% CI, 0.63–0.79]; **Figure 2**). The 5-year OS of the entire TriNetX esophageal cancer male cohort is 44.6% (N = 83,839).

### Laryngeal cancer

Post‐PSM, the PDE5 inhibitor and control cohorts each included 1,116 patients with laryngeal cancer (**Tables 4**, **5**). PDE5 inhibitor exposure was associated with improved OS at all timepoints compared with controls (1‐year OS 92.5% vs 85.0%, p<0.001; 3‐year OS 84.1% vs 73.3%, p<0.001; 5‐year OS 76.9% vs 65.8%, p<0.001; HR, 0.60 [95% CI, 0.49–0.72]; **Figure 2**). The 5-year OS of the entire TriNetX laryngeal cancer male cohort is 65.9% (N = 62,015).

### Tonsillar cancer

Post‐PSM, the PDE5 inhibitor and control cohorts each included 1,211 patients with tonsillar cancer (**Tables 4**, **5**). PDE5 inhibitor exposure was associated with significantly improved OS at all timepoints compared with controls (1‐year OS 96.2% vs 90.7%, p<0.001; 3‐year OS 90.7% vs 81.0%, p<0.001; 5‐year OS 86.1% vs 77.5%, p<0.001; HR, 0.56 [95% CI, 0.44–0.71]; **Figure 2**). The 5-year OS of the entire TriNetX tonsillar cancer male cohort is 76.7% (N = 35,576).

### Testicular cancer

Post‐PSM, the PDE5 inhibitor and control cohorts each included 1,326 patients with testicular cancer (**Tables 4**, **5**). PDE5 inhibitor exposure was associated with significantly improved OS at all timepoints compared with controls (1‐year OS 98.3% vs 94.5%, p<0.001; 3‐year OS 94.9% vs 90.2%, p<0.001; 5‐year OS 92.2% vs 89.2%, p=0.003; HR, 0.63 [95% CI, 0.47–0.86]; **Figure 2**). The 5-year OS of the entire TriNetX testicular cancer male cohort is 91.3% (N = 50,737).

### Hypopharyngeal cancer

Post‐PSM, the PDE5 inhibitor and control cohorts each included 213 patients with hypopharyngeal cancer (**Tables 4**, **5**). PDE5 inhibitor exposure was associated with significantly improved OS at all timepoints compared with controls (1‐year OS 87.4% vs 76.4%, p=0.004; 3‐year OS 69.9% vs 58.2%, p=0.008; 5‐year OS 63.9% vs 48.0%, p=0.002; HR, 0.59 [95% CI, 0.42–0.82]; **Figure 2**). The 5-year OS of the entire TriNetX hypopharyngeal cancer male cohort is 56.6% (N = 16,042).

## Discussion

This study provides population-level, propensity score-matched data evaluating the association between PDE5 inhibitor exposure and OS in male-predominant cancers. In a cohort of 217,260 matched male patients drawn from a large multi-institutional database, PDE5 inhibitor use was associated with an overall survival advantage across all stages and cancer types at all timepoints investigated. This analysis represents, to the best of our knowledge, the first pan-cancer evaluation of PDE5 inhibitors in male-predominant tumors, expanding upon prior site-specific observations in colorectal, prostate, and gastric cancers. By demonstrating a survival advantage not only in genitourinary and gastrointestinal cancers, but also in head and neck subsites where prior work has been limited to immune modulation rather than survival outcomes, this study suggests a potentially generalizable oncologic benefit of PDE5 inhibitors and highlights a widely prescribed, well-tolerated drug class as a candidate for repurposing in oncology. Restricting the analysis to male-predominant cancers was intentional to minimize sex-based confounding and to focus on populations where PDE5 inhibitor exposure is clinically common. These tumor types also share relevant clinical and epidemiologic features that help reduce heterogeneity when assessing post-diagnosis PDE5 inhibitor exposure. While analyses of sex-balanced or female-predominant cancers were beyond the present scope, future studies may explore whether these associations extend to broader oncologic populations.

The survival advantage observed in our analysis extends the emerging body of evidence supporting an oncologic role for PDE5 inhibitors. In a cohort of 161 patients with gastric cancer, Zhang et al. reported lower cancer-specific mortality among PDE5 inhibitor users (adjusted HR 0.66, 95% CI 0.47-0.92). This clinical observation was supported by mechanistic studies in gastric cancer cell lines, tumor organoids, and xenograft models, demonstrating that sildenafil suppresses tumor growth by inhibiting PDE5, destabilizing c-MYC, and downregulating IL-6/JAK/STAT3 signaling (16). Consistent with this evidence, Huang et al. studied a nationwide cohort of 12,465 male patients with stage I-III colorectal cancer and found that post-diagnosis PDE5 inhibitor use was associated with a reduction in cancer-specific mortality and metastatic progression, with the strongest protective effect observed in those treated after open surgery (adjusted HR 0.82, 95% CI 0.67-0.99; adjusted HR 0.85, 95% CI 0.74-0.98, respectively) (17). In addition, in a propensity score-matched analysis of 1,058 patients undergoing robot-assisted radical prostatectomy, Lee et al. reported that PDE5 inhibitor use was associated with a lower mortality risk without an increase in cancer recurrence (18). Taken together, prior studies provide the mechanistic and epidemiologic foundation for an oncologic role of PDE5 inhibitors, which our work extends across additional male-predominant cancer sites including prostate, bladder, laryngeal, and esophageal. Importantly, these mechanistic data provide biological plausibility but do not establish causality in the context of our observational findings. Thus, the associations reported here should be interpreted as hypothesis-generating rather than evidence of a direct therapeutic effect.

The association observed in our study is consistent with preclinical evidence demonstrating immunomodulatory effects of PDE5 inhibition within the tumor microenvironment (8, 14). Specifically, PDE5 inhibition downregulates arginase-1 and nitric oxide synthase in myeloid-derived suppressor cells (MDSCs), thereby impairing their suppressive function and facilitating cytotoxic T-cell infiltration (8, 19). In murine tumor models, PDE5 inhibition slows tumor growth and enhances antitumor immune responses (10). Clinical studies in patients with head and neck squamous cell carcinoma have shown that tadalafil reduces circulating MDSCs and increases tumor-specific immune activation (20, 21). Moreover, in multiple preclinical models, including melanoma, pancreatic adenocarcinoma, and hepatocellular carcinoma, PDE5 inhibitors have demonstrated synergy with immune checkpoint blockade, further supporting their potential as immunomodulatory adjuncts in cancer therapy (14, 15, 22).

Clinically, these findings support the rationale for considering PDE5 inhibitors as adjuncts in oncologic therapy. These agents are inexpensive, orally administered, and have well-established safety profiles in men, making them potential candidates for therapeutic repurposing. Nonetheless, PDE5 inhibitors are not without contraindications, particularly in patients with cardiovascular instability or nitrate use, and their safety in cancer populations has not been fully characterized. Observational data also suggest that perioperative PDE5 inhibitor use may reduce the pro-metastatic effects of surgical stress by limiting the expansion of immunosuppressive myeloid cells, further supporting their role alongside other cancer treatments (23). However, given the absence of randomized clinical trials evaluating PDE5 inhibitors for cancer outcomes, these observational associations should not be taken to imply clinical benefit and should be interpreted cautiously.

This study has several limitations. Despite propensity score matching across demographic, oncologic, and comorbidity variables, residual confounding remains possible given the lack of data on performance status, treatment intensity, and socioeconomic context. Due to limited sample sizes, stage-stratified analyses were only feasible in the combined pan-cancer cohort and may not fully capture survival outcomes within each individual cancer type. Reliance on TriNetX data restricted our analyses to overall survival, as standardized progression or recurrence events are not uniformly captured across contributing institutions in TriNetX, precluding a reliable progression-free and disease-free survival analyses. Exposure was defined by any PDE5 inhibitor prescription within 6 months of diagnosis; adherence, dose, duration, and agent-specific effects were not available, and we could not distinguish short-term or perioperative use from sustained therapy. This approach may capture heterogeneous usage patterns, including peri-diagnostic or short-term prescriptions, and may introduce exposure misclassification. Treatment-level data, including surgery, radiation, chemotherapy, and immunotherapy, were variably captured across institutions in TriNetX and were not uniformly available. The platform does not support user-defined multivariable or Cox regression analyses beyond built-in Kaplan–Meier and hazard ratio tools; thus, accounting for these variables was not feasible and remains an acknowledged limitation. Because patients must survive long enough after diagnosis to receive a prescription, some degree of immortal-time bias is possible. The particularly strong association observed in prostate cancer may partly reflect channeling bias, as younger or healthier men are more likely to receive PDE5 inhibitors. Additionally, the TriNetX population may not reflect the exact survival trends of the general population in the examined cancers. To provide context, we reported 5-year overall survival estimates from all TriNetX patients of each included cancer type. These values differ from those published in population-based datasets, reflecting possible differences in participating institutions, referral patterns, patient health-seeking behavior, and data-capture methods (24, 25). Lastly, this analysis was restricted to male patients with cancers showing male predominance to reduce sex-based confounding and reflect the population most likely to receive PDE5 inhibitors. As such, the findings may not generalize to sex-balanced or female-predominant cancers, which warrant dedicated evaluation.

Future research should focus on prospective randomized trials to determine whether PDE5 inhibitors can improve outcomes when added to standard cancer treatments, especially in combination with established immunotherapy regimens. These studies should go beyond survival data and explore biomarkers that reflect immune activation and tumor response. Additionally, such studies could prioritize cancer types with the strongest observed associations (e.g., prostate, colorectal), as well as advanced-stage disease where immunomodulation may be most impactful. It will also be important to clarify when and how these drugs are most effective, whether given around the time of surgery, as ongoing therapy, or alongside immunotherapy.

## Conclusion

Using a real-world global database, population-level data, this large cohort study found that PDE5 inhibitor use is associated with improved 1-, 3-, and 5-year overall survival in male patients with male-predominant solid tumors. These findings support further investigation into the immunologic and therapeutic effects of PDE5 inhibitors in oncology.

## Data Availability

TriNetX data are available to qualified researchers via institutional agreements with TriNetX and cannot be publicly shared by the authors. SEER data are publicly available. Requests to access these datasets should be directed to https://live.trinetx.com.

## References

[1] Lasser SA Ozbay Kurt FG Arkhypov I Utikal J Umansky V . Myeloid-derived suppressor cells in cancer and cancer therapy. Nat Rev Clin Oncol. (2024) 21:147–64. doi: 10.1038/s41571-023-00846-y, PMID: 38191922

[2] Chen H Xu Z Varner J . Targeting myeloid cells to improve cancer immune therapy. Front Immunol. (2025) 16:1623436. doi: 10.3389/fimmu.2025.1623436, PMID: 40821795 PMC12350267

[3] Zhao Y Du J Shen X . Targeting myeloid-derived suppressor cells in tumor immunotherapy: Current, future and beyond. Front Immunol. (2023) 14:1157537. doi: 10.3389/fimmu.2023.1157537, PMID: 37006306 PMC10063857

[4] Bray F Laversanne M Sung H Ferlay J Siegel RL Soerjomataram I . Global cancer statistics 2022: GLOBOCAN estimates of incidence and mortality worldwide for 36 cancers in 185 countries. CA A Cancer J Clin. (2024) 74:229–63. doi: 10.3322/caac.21834, PMID: 38572751

[5] Gabrilovich DI Nagaraj S . Myeloid-derived suppressor cells as regulators of the immune system. Nat Rev Immunol. (2009) 9:162–74. doi: 10.1038/nri2506, PMID: 19197294 PMC2828349

[6] Rodriguez PC Zea AH DeSalvo J Culotta KS Zabaleta J Quiceno DG . l -arginine consumption by macrophages modulates the expression of CD3ζ Chain in T lymphocytes. J Immunol. (2003) 171:1232–9. doi: 10.4049/jimmunol.171.3.1232, PMID: 12874210

[7] Andersson K . PDE5 inhibitors – pharmacology and clinical applications 20 years after sildenafil discovery. Br J Pharmacol. (2018) 175:2554–65. doi: 10.1111/bph.14205, PMID: 29667180 PMC6003652

[8] Serafini P Meckel K Kelso M Noonan K Califano J Koch W . Phosphodiesterase-5 inhibition augments endogenous antitumor immunity by reducing myeloid-derived suppressor cell function. J Exp Med. (2006) 203:2691–702. doi: 10.1084/jem.20061104, PMID: 17101732 PMC2118163

[9] Noonan KA Ghosh N Rudraraju L Bui M Borrello I . Targeting immune suppression with PDE5 inhibition in end-stage multiple myeloma. Cancer Immunol Res. (2014) 2:725–31. doi: 10.1158/2326-6066.CIR-13-0213, PMID: 24878583 PMC4152913

[10] Califano JA Khan Z Noonan KA Rudraraju L Zhang Z Wang H . Tadalafil augments tumor specific immunity in patients with head and neck squamous cell carcinoma. Clin Cancer Res. (2015) 21:30–8. doi: 10.1158/1078-0432.CCR-14-1716, PMID: 25564570 PMC4329916

[11] Luginbuhl AJ Johnson JM Harshyne LA Linnenbach AJ Shukla SK Alnemri A . Tadalafil enhances immune signatures in response to neoadjuvant nivolumab in resectable head and neck squamous cell carcinoma. Clin Cancer Res. (2022) 28:915–27. doi: 10.1158/1078-0432.CCR-21-1816, PMID: 34911681 PMC8898272

[12] Serafini P Borrello I Bronte V . Myeloid suppressor cells in cancer: Recruitment, phenotype, properties, and mechanisms of immune suppression. Semin Cancer Biol. (2006) 16:53–65. doi: 10.1016/j.semcancer.2005.07.005, PMID: 16168663

[13] Sutton SS Magagnoli J Cummings TH Hardin JW . The association between phosphodiesterase-5 inhibitors and colorectal cancer in a national cohort of patients. Clin Transl Gastroenterol. (2020) 11:e00173. doi: 10.14309/ctg.0000000000000173, PMID: 32568473 PMC7339197

[14] Gross NE Zhang Z Mitchell JT Charmsaz S Hernandez AG Coyne EM . Phosphodiesterase-5 inhibition collaborates with vaccine-based immunotherapy to reprogram myeloid cells in pancreatic ductal adenocarcinoma. JCI Insight. (2024) 9:e179292. doi: 10.1172/jci.insight.179292, PMID: 39106104 PMC11457845

[15] Wang X Zhang Q Zhou J Xiao Z Liu J Deng S . T cell-mediated targeted delivery of tadalafil regulates immunosuppression and polyamine metabolism to overcome immune checkpoint blockade resistance in hepatocellular carcinoma. J Immunother Cancer. (2023) 11:e006493. doi: 10.1136/jitc-2022-006493, PMID: 36813307 PMC9950981

[16] Zhang Z Huang W Huang D Xu Z Xie Q Tan X . Repurposing of phosphodiesterase-5 inhibitor sildenafil as a therapeutic agent to prevent gastric cancer growth through suppressing c-MYC stability for IL-6 transcription. Commun Biol. (2025) 8:85. doi: 10.1038/s42003-025-07519-9, PMID: 39827331 PMC11742916

[17] Huang W Sundquist J Sundquist K Ji J . Phosphodiesterase-5 inhibitors use and risk for mortality and metastases among male patients with colorectal cancer. Nat Commun. (2020) 11:3191. doi: 10.1038/s41467-020-17028-4, PMID: 32581298 PMC7314744

[18] Lin S Wang J Wang L Wen J Guo Y Qiao W . Phosphodiesterase-5 inhibition suppresses colonic inflammation-induced tumorigenesis via blocking the recruitment of MDSC. Am J Cancer Res. (2017) 7:41–52. 28123846 PMC5250679

[19] Weed DT Vella JL Reis IM de la Fuente AC Gomez C Sargi Z . Tadalafil reduces myeloid-derived suppressor cells and regulatory T cells and promotes tumor immunity in patients with head and neck squamous cell carcinoma. Clin Cancer Res. (2015) 21:39–48. doi: 10.1158/1078-0432.CCR-14-1711, PMID: 25320361 PMC4322895

[20] Tai LH Alkayyal AA Leslie AL Sahi S Bennett S Tanese De Souza C . Phosphodiesterase-5 inhibition reduces postoperative metastatic disease by targeting surgery-induced myeloid derived suppressor cell-dependent inhibition of Natural Killer cell cytotoxicity. OncoImmunology. (2018) 7:e1431082. doi: 10.1080/2162402X.2018.1431082, PMID: 29872554 PMC5980420

[21] Lee J Kim HR Heo JE Jang WS Lee KS Kang SK . Phosphodiesterase-5 inhibitor use in robot assisted radical prostatectomy patients is associated with reduced risk of death: A propensity score matched analysis of 1,058 patients. World J Mens Health. (2023) 41:892. doi: 10.5534/wjmh.220063, PMID: 36649919 PMC10523119

[22] Hassel JC Jiang H Bender C Winkler J Sevko A Shevchenko I . Tadalafil has biologic activity in human melanoma. Results of a pilot trial with Ta dalafil in patients with metastatic Melanoma (TaMe). OncoImmunology. (2017) 6:e1326440. doi: 10.1080/2162402X.2017.1326440, PMID: 28932631 PMC5599085

[23] Pantziarka P Sukhatme V Crispino S Bouche G Meheus L Sukhatme VP . Repurposing drugs in oncology (ReDO)—selective PDE5 inhibitors as. ecancer (2018). Available online at: https://www.ecancer.org/journal/12/full/824-repurposing-drugs-in-oncology-redo-selective-pde5-inhibitors-as-anti-cancer-agents.php (Accessed September 9, 2025). 10.3332/ecancer.2018.824 PMC5931815 29743944

[24] National Cancer Institute . Surveillance, Epidemiology, and End Results (SEER) Program . National Cancer Institute. Available online at: https://seer.cancer.gov (Accessed September 19, 2025).

[25] National Cancer Institute . National Cancer Care Registry (NCCR) . National Cancer Institute. Available online at: https://nccr.cancer.gov (Accessed September 19, 2025).

